# Effectiveness of antiviral, immunomodulatory and platelet-enhancing agents for treatment of dengue infection: A systematic review

**DOI:** 10.1080/21505594.2025.2587491

**Published:** 2025-11-10

**Authors:** D.G. Aynekulu Mersha, F. Duijff, T. Langerak, M.S. Hakim, B. Martina, M. Goeijenbier, V.A.S.H. Dalm, S.F.L. van Lelyveld, E.C.M. van Gorp

**Affiliations:** a Department of Viroscience, Erasmus University Medical Center, Rotterdam, The Netherlands; b Department of Internal Medicine, Ikazia Hospital, Rotterdam, The Netherlands; c Postgraduate School of Molecular Medicine, Erasmus University Medical Center, Rotterdam, The Netherlands; d Department of Biology and Immunology, College of Medicine, Qassim University, Buraydah, Saudi Arabia; e Artemis Bioservices and Athenavax B.V, Delft, The Netherlands; f Department of Intensive Care, Spaarne Gasthuis, Haarlem/Hoofddorp, The Netherlands; g Division of Allergy and Clinical Immunology, Department of Internal Medicine, and Department of Immunology, Erasmus University Medical Center, Rotterdam, The Netherlands; h Department of Internal Medicine, Spaarne Gasthuis, Haarlem/Hoofddorp, The Netherlands

**Keywords:** Dengue, treatment, antiviral, immunity, platelet

## Abstract

Dengue is a vector-borne infectious disease, caused by dengue virus (DENV), with a rapidly increasing incidence worldwide. With no feasible, widely applicable prevention method available in the near term, the need for an effective treatment is of great importance. This systematic review aims to provide a comprehensive overview of potential antiviral, immunomodulatory, and platelet-enhancing therapies for the treatment of DENV. This systematic review was conducted according to the PRISMA guidelines. Clinical trials that investigate treatment options for DENV in the general population were included. Twenty-six studies were included, investigating length of hospital stay (*n* = 10), platelet count (*n* = 16), interleukin (IL)-6 levels (*n* = 4), virological log reduction (VLR) (*n* = 2), and non-structural (NS)-1 clearance time (*n* = 4). Focusing on potential antiviral agents, four studies showed a significant reduction regarding length of hospital stay, of which two used doxycycline. The most profound reduction of hospital stay was observed when doxycycline was combined with *Carica papaya* herbal extract (7.3 days vs 9.1 days). This combination was also able to achieve a significant rise in platelet count (+154.1 × 10^9^/L vs + 66.0 × 10^9^/L in 7 days). Immunomodulatory therapies did not demonstrate efficacy against DENV, although some evidence suggests that rupatadine may increase platelet count. The platelet-enhancing agents recombinant human IL-11, anti-rhD immunoglobulin (anti-D), and eltrombopag all showed a significant rise in platelet count. Small sample sizes make it challenging to draw definitive conclusions out of the included studies. Larger clinical trials are needed to evaluate treatments for DENV, with particular focus on doxycycline, *Carica papaya*, rupatadine, and platelet-enhancing agents.

## Introduction

In 2023 over six million cases of dengue occurred worldwide and almost 7000 deaths were reported making it the most widespread vector-borne viral infectious disease worldwide [1,2]. Unfortunately, the trend continued into 2024, with over 13 million dengue cases reported by September [3]. The four dengue virus serotypes (DENV1-4) circulate together in tropical and subtropical areas, causing half of the world’s population being at risk for DENV [4,5].

This large part of the globe being at risk for an infection becomes even of more importance when taking into account that, one DENV serotype is thought to confer long lasting immunity only against that specific serotype. Against the other serotypes only temporary protection occurs [1]. The majority of dengue-related infections are mild or asymptomatic. However, some develop a more serious infection entering a critical phase, characterized by plasma leakage and hemorrhagic complications, more commonly seen in secondary infections [6]. These DENV infections are categorized based on symptoms, aligning with the 2009 World Health Organization (WHO) classification as dengue with or without warning signs and severe dengue (SD) [7]. Nonetheless, the 1997 WHO guideline remains widely used in practice, classifying infections as dengue hemorrhagic fever (DHF) or dengue shock syndrome (DSS).

This more severe course of disease during secondary infections is suggested to be due to antibody dependent enhancement (ADE), which is an immune-pathological phenomenon associated with enhanced disease severity. It occurs due to inadequate heterotypic immunity when an individual with circulating antibodies against one DENV serotype is subsequently infected with a different DENV serotype. These antibodies are cross-reactive, but lack sufficient neutralizing activity, or are present in insufficient concentrations. Antibodies can enhance DENV entry into cells, enabling viral replication and potentially initiating a pathological cascade that leads to severe dengue [8]. Moreover, SD can also occur during primary infections, more often seen in children under the age of one year and in immunosurpressed individuals [9,10]

The presence of ADE complicates the development of effective prevention strategies for DENV. While extensive research is being conducted in the field of vector control, vaccine development has also proven challenging [11]. This is driven by the need for effectiveness against all serotypes while avoiding the risk of ADE [12]. The first registered vaccine initially met primary outcomes in efficacy trials [13]. However, a long-term follow-up study revealed more hospital admissions among seronegative children under the age of nine [14]. Since 2022, a second vaccine has been available on the market, showing a 4,5-year cumulative efficacy of 61.2% against virologically confirmed dengue [15,16]. However, this vaccine is predominantly administered only to seropositives.

With no feasible, widely applicable prevention method available in the near term to address the global dengue burden, an urgent need to develop effective treatment options maintains. Various components of DENV during the different stages of its life cycle are considered potential targets for the development of antiviral agents [17]. Yet, no therapeutic agent is officially licensed for use.

Considering the limited availability of effective preventive strategies, the potential of therapies for combating DENV is discussed in this systematic review. The objective is to provide an overview of potential treatments for DENV and to identify the most promising therapeutic agents for future research.

## Methods

### Study selection

Medline, Embase, Web of Science, and Cochrane were searched from their inception up to 2 September 2024. The search terms used were: “dengue,” “therapy,” “anti-infective,” “anti-viral,” “antibiotic,” “anti-parasitic,” “ivermectin,” “doxycycline,” “chloroquine,” “oseltamivir,” “lovastatin.” The search strings and their corresponding results can be found in Supplementary File 1.

Article selection was conducted by two independent reviewers (D.A.M. and F.D.). Initially, articles were screened based on their titles and abstracts. Secondly, eligible articles were included after an examination of the full texts. Any disagreements at any stage were resolved through discussion and consensus. The inclusion and assessment of the articles were conducted according to PRISMA guidelines [18]. No ethical approval or informed consent was required for this systematic review.

### Inclusion and exclusion criteria

Studies included in this review cover clinical trials that investigate the effectiveness of pharmacological interventions with theoretical or demonstrated antiviral properties, or with immunomodulatory, or with platelet improving abilities. All studies investigating supportive treatment with no known or hypothesized antiviral, immunomodulatory or platelet-enhancing mechanism as an intervention were excluded. Examples are platelet transfusion, vitamin supplementation or various rehydration therapies.

Patients included in this review all had a confirmed DENV infection, either confirmed by PCR, IgM antibodies or NS1 antigen. In vitro studies, non-human studies and studies involving healthy subjects (phase 1 studies) were excluded. Studies with a total sample size of less than 25 subjects where excluded. With the impossibility of performing a meta-analysis, no random effects model could be used to interpret the results of very small studies with caution. Therefore, this benchmark of 25 patients was chosen pragmatically to avoid the augmented chance of very small studies, potentially suffering from publication bias, to change the interpretation of the narrative synthesis. To reduce clinical heterogenity, studies solely investigating a pediatric study population were also excluded. This was due to the differences in both the clincial manifestations, immunological differences, as possible differences in outcome of treatment in a pediatric population [19,20]. Only studies published in English were included. If studies were not available in full text, efforts where made to contact the corresponding author via mail, to a maximum of three attempts. If no response was given two weeks after the last mailing approach, the article was excluded from the analysis.

### Quality assessment

The methodological quality of the evidence of the included randomized controlled trials (RCTs) was evaluated using the Cochrane Risk of Bias tool [21], classifying articles into “high risk of bias,” “low risk of bias,” or “unclear risk of bias.” Studies that did not use randomization were evaluated using the ROBINS-1 tool [22]. With this tool, different domains were assessed and categorized as “low risk of bias,” “moderate risk of bias,” “serious risk of bias,” “critical risk of bias,” or “no information.”

### Outcomes

The two primary outcomes examined are the length of hospitalization and improvement in platelet count during admission. Hospital stay is stated as days admitted. For platelet count, endpoint measurements were collected and are stated as amount *10^9^/L. The endpoint for platelet count is defined by the number of days after admission, with baseline considered as day 0. Secondary outcomes analyzed are the value of interleukin-6 (IL-6), the Nonstructural protein 1 (NS1) clearance time, and the virological log reduction (VLR) per day. For IL-6, data were collected for the endpoint at which IL-6 levels were determined in pg/ml. The endpoint for IL-6 is also specified as the number of days after admission, with baseline considered as day 0. The NS1 clearance time is, if necessary, transformed in hours. VLR is stated as log_10_ copies/mL/day. Continuous variables were stated as means with their 95% confidence intervals (CIs) or as medians with their corresponding interquartile ranges (IQRs). Categorical variables are stated as proportions with their corresponding CIs.

### Data extraction and analysis

Data was extracted using Covidence, an online application for screening of references and undertaking data extraction. The study characteristics included the name of the first author, year of publication, location, study design, sample size, key inclusion criteria, interventions used, outcomes, and the method of outcome reporting. When raw data were available after contacting corresponding authors and essential analyses relevant to this review were missing, the analyses were conducted independently by the authors of this review (both D.A.M and F.D). The results were synthesized narratively. Figures regarding the quality assessment were made using RStudio version 2024.04.2

## Results

### Search results

**Figure 1.**
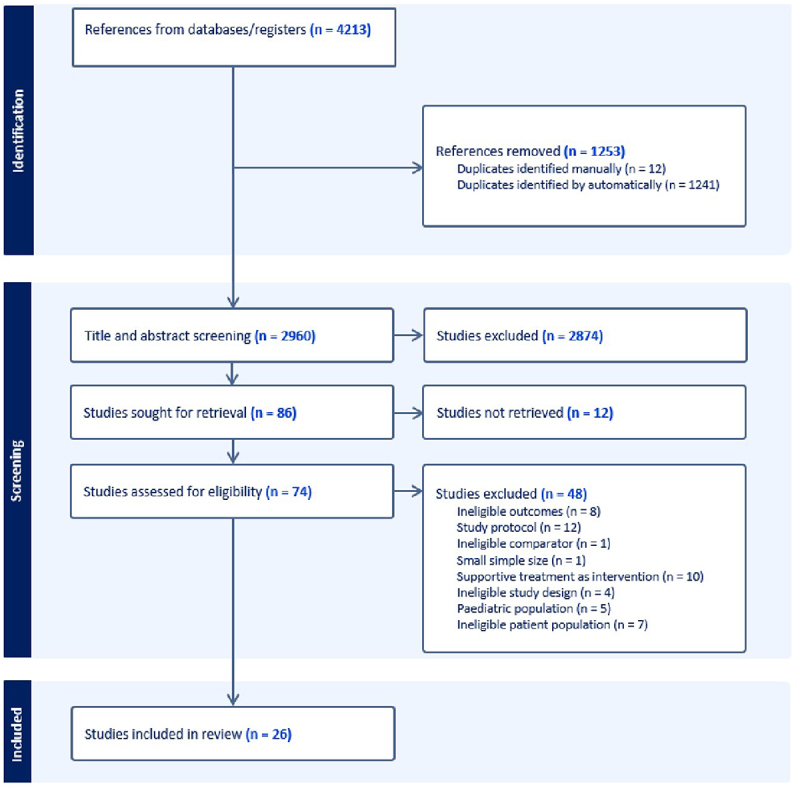
Flowchart of screening process and study selection.

The flowchart of the study selection is depicted in Figure 1. The search strategy described in the method section yielded 3495 references, after which duplications were removed. After the initial screening of the studies, 86 remained sought for retrieval. 74 studies were available in full text and therefore, assessed for eligibility. 48 studies were excluded for various reasons, based on aforementioned criteria. 26 studies remained eligible for inclusion [23–48].

### Quality assessment

The quality assessment of all included articles is depicted in Figures 2 and 3. Of the RCTs, four articles scored an overall low risk of bias [31,34,43,44], seven articles a moderate risk of bias [28,32,35–37,41,48], and eleven articles a high risk of bias [23–25,27,29,33,39,42,45–47]. Selective outcome reporting was unclear for almost all articles, due to the small amount of papers providing information about the possibility of selective outcome reporting. Therefore, this domain is not considered when assessing the overall risk of bias. Small sample sizes accounted for the majority of “high” scores in the “other sources of bias” domain, followed by discrepancies in baseline characteristics. Due to small sample sizes in a large number of studies, this domain was likewise excluded in the overall risk of bias assessment.

**Figure 2A.**
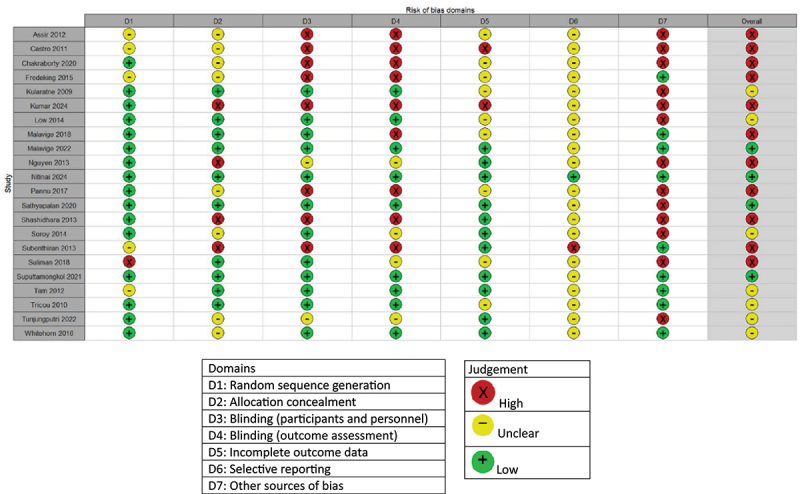
Quality assessment of the included RCT’s.

**Figure 2B.**
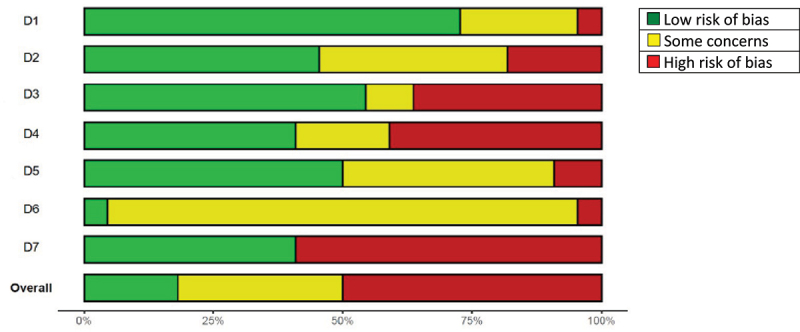
Overall quality of the included RCT’s by domain.

**Figure 3.**
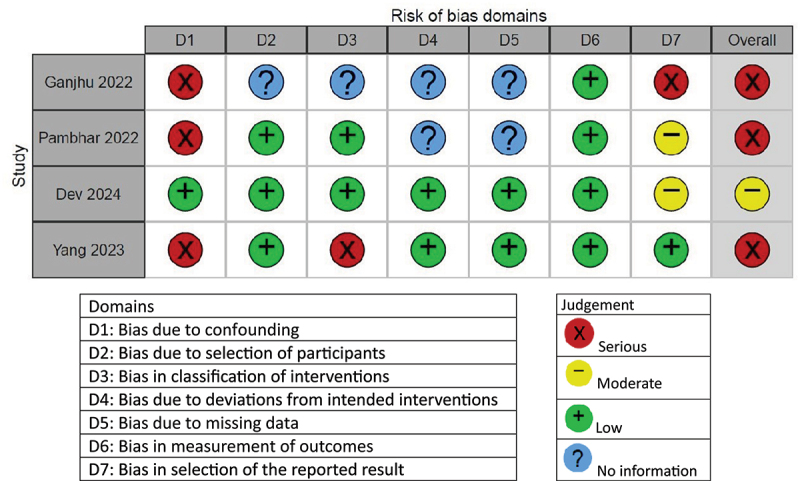
Quality assessment of included non-randomized trials.

Four articles were assessed according to the ROBINS-1 tool [26,30,38,40], of which three had an overall serious risk of bias [26,30,38], and one had an overall moderate risk of bias [40]. The quality assessment of the non-randomized trials is depicted in Figure 3.

### Study characteristics

A summary of the included studies is presented in Tables 1 and 2. The studies were conducted between 2009 and 2024 in various countries across Asia and Latin America. All studies focused on hospitalized patients, except for two, which were conducted in hospital’s outpatient clinics [43,44]. The majority of the studies were RCTs, with the exception of four studies that were observational in design [26,30,38,40].

**Table 1.** Study characteristics of studies focusing on antiviral treatment.

Author	Year	Location	Study design	Sample size	Patient population	Intervention	Outcomes
Assir et al. [20]	2012	Pakistan	Randomized controlled trial	39	DF + thrombocytopenia or DHF + thrombocytopenia	Carica papaya	Platelet count
Castro et al. [21]	2011	Latin America	Randomized controlled trial	114	DF or DHF	Doxycycline or tetracycline	IL-6
Fredeking et al. [22]	2015	Mexico	Randomized controlled trial	231	DHF	Doxycycline	Hospital stay + IL-6
Ganjhu [23]	2022	India	Case- control study	54	DF + thrombocytopenia	Ganjhuvirr	Hospital stay + platelet count
Kumar et al. [24]	2024	India	Randomized controlled trial	69	DF	Doxycycline	Platelet count + IL-6
Low et al. [25]	2014	Singapore	Randomized controlled trial	69	DF	Celgosivir	Viral log reduction
Nguyen et al. [26]	2013	Vietnam	Randomized controlled trial	64	DF	Balapiravir	NS1 clearance time
Pambhar et al. [27]	2022	India	Case-control study	120	DF + thrombocytopenia	Doxycycline or doxycycline + carica papaya	Hospital stay + platelet count
Sathyapalan et al. [28]	2020	India	Randomized controlled trial	51	DF + thrombocytopenia	Carica papaya	Hospital stay + platelet count + IL-6
Soroy et al. [29]	2014	Indonesia	Randomized controlled trial	63	DHF	Propoelix^TM^	Hospital stay + platelet count
Subenthiran et al. [30]	2013	Malaysia	Randomized controlled trial	290	DF or DHF	Carica papaya	Platelet count
Suputtamongkol et al. [31]	2021	Thailand	Randomized controlled trial	206	DF	Ivermectin	Hospital stay + viral log reduction + NS1 clearance time
Tricou et al. [32]	2010	Vietnam	Randomized controlled trial	307	DF	Chloroquine	NS1 clearance time
Tunjungputri et al. [33]	2022	Indonesia	Randomized controlled trial	70	DF + thrombocytopenia	Oseltamivir	Platelet count
Whitehorn et al. [34]	2016	Vietnam	Randomized controlled trial	300	DF	Lovastatin	Hospital stay
Yang et al. [35]	2023	China	Retrospective cohort study	274	DF	CSJD granules	Platelet count

**Table 2.** Study characteristics of studies focusing on immunomodulatory or platelet improving treatment.

Author	Year	Location	Study design	Sample size	Patient population	Intervention	Outcomes
Chakrabotry et al. [36]	2020	Bangladesh	Randomized controlled trial	109	DF + thrombocytopenia	Eltrombopag	Platelet count
Dev et al. [37]	2024	India	Prospective cohort study	500	DF	Montelukast	Hospital stay
Kularatne et al. [38]	2009	Sri Lanka	Randomized controlled trial	200	DF + thrombocytopenia	Dexamethasone	Platelet count
Malavige et al. [39]	2018	Sri Lanka	Randomized controlled trial	133	DF	Rupatadine	Platelet count
Malavige et al. [40]	2022	Sri Lanka	Randomized controlled trial	271	DF	Rupatadine	Platelet count
Nitinai et al. [41]	2024	Thailand	Randomized controlled trial	357	DF	Montelukast	Hospital stay
Pannu et al. [42]	2017	India	Randomized controlled trial	30	DF* + thrombocytopenia	Anti-D	Hospital stay + platelet count
Suliman et al. [43]	2014	Pakistan	Randomized controlled trial	40	DF + thrombocytopenia	rhIL-11	Platelet count
Shashidhara et al. [44]	2013	India	Randomized controlled trial	61	DF + thrombocytopenia	Dexamethasone	Platelet count
Tam et al. [45]	2012	Vietnam	Randomized controlled trial	225	DF	Prednisolone	NS1 clearance time

Abbreviations: DF = dengue fever; DHF = dengue hemorrhagic fever; IL-6 = interleukin-6; NS1 = nonstructural protein 1; CSJD = Chai-Shi-Jie-Du; rhIL-11 = recombinant human interleukin-11. *Patient population consisted of rhesus positive patients.

Regarding studies with antiviral agents, four used doxycycline as the intervention [24,25,27,30]. Others used different antimicrobial [24], antiviral [28,29,36], antiparasitic [34], or antimalarial [35] regimens. Multiple studies used pharmacological agents derived from herbal extracts [23,26,30–33,38]. The remaining article used a statin [37].

Among the studies on immunomodulation, three examined corticosteroids [41,47,48], while other studies assessed the effects of the antihistamines montelukast [40,44], and rupatadine [42,43]. Three studies evaluated platelet-enhancing agents, including eltrombopag [39], anti-D [45], and recombinant human interleukin-11 (rhIL-11) [46].

The majority of the studies included patients with either DF or DF combined with thrombocytopenia. One study [32] included only DHF patients and two studies [24,33] included both DF patients and DHF patients. Assir *et al*. [23] focused on DENV-associated thrombocytopenia in patients with either DF or DHF.

### Agents with antiviral properties

#### Length of hospital stay

Seven articles reported data on the length of hospital stay, which are depicted in Table 3 [22,23,27–30,33]. Two studies, investigating the intervention in DHF, showed a significant difference between the intervention (doxycycline or propoelix^TM^) and the control group [25,32]. Two other studies, investigating ganjhuvirr, doxycycline, and *Carica papaya*, also showed a significant difference [26,30]. These studied patients with DF combined with thrombocytopenia. The difference in mean hospital stay described by Ganjhu *et al*. is striking, with 3.65 ± 0.97 days in the ganjhuvirr group and 6.20 ± 0.98 days in the control group (*p* < 0.01) [26]. Other studies found no significant difference in the duration of hospital stay between the intervention and control groups when treated with *Carica papaya*, ivermectin, or lovastatin, respectively [31,34,37].

**Table 3.** Data on length of hospital stay (in days).

Study	Inclusion criteria	Intervention	Outcome reported as	Result (intervention vs control)
Fredeking 2015 [22]	DHF	Doxycycline	Mean	6.8* vs 7.87
Ganjhu 2022 [23]	DF + thrombocytopenia	Ganjhuvirr	Mean ± SD	3.65* ± 0.97 vs 6.20 ± 0.98
Pambhar 2022 [27]	DF + thrombocytopenia	Doxycyline + carica papaya or doxycycline	Mean	7.3* vs 8.2 vs 9.1
Sathyapalan 2020 [28]	DF + thrombocytopenia	Carica papaya	Mean ± SD	5 ± 1 vs 5 ± 1
Soroy 2014 [29]	DHF	Propoelix^TM^	Mean ± SD	4.69* ± 0.78 vs 5.46 ± 1.16
Suputtamongkol 2021 [31]	DF	Ivermectin	Mean ± SD	5.3 ± 1.6 vs 5.2 ± 1.2
Whitehorn 2016 [34]	DF	Lovastatin	Median (IQR)	8 [7,8] vs 8 [7,8]

*Significant value (P-value < 0.05).

Abbreviations: DHF = dengue hemorrhagic fever; DF = dengue fever; SD = standard deviation; IQR = interquartile range.

Both studies with doxycycline as an intervention showed a shorter length of hospital stay [25,30]. Furthermore, *Carica papaya* in combination with doxycycline resulted in a shorter mean length of hospital stay of 7.3 days, compared to both doxycycline alone (8.2 days; *p* < 0.01) and the control group (9.1 days; *p* = 0.01) [30]. However, Sathyapalan *et al*. could not find a significant difference with the use of *Carica papaya* alone in patients with DF and thrombocytopenia [31].

#### Platelet count

Outcome characteristics and results of the nine articles regarding platelet count are presented in Table 4 [23,26,27,30–33,36,38]. All studies included patients with thrombocytopenia. Half of the studies showed a significantly higher platelet rise in count of the intervention compared to the control group at the endpoint [26,30–32]. Interestingly, all of these studies used a form of herbal extracts, although two other studies that investigated the effect of *Carica papaya*, and one study evaluating Chai-Shi-Jie-Du (CSJD) granules showed no significant difference in platelet count [23,33,38].

**Table 4.** Outcome characteristics of platelet count (number ×10^9^/L).

Study	Patient population	Intervention	Reported as	Time of endpoint (in days)	Change from baseline	Value at endpoint
Assir 2012 [20]	DF + thrombocytopenia or DHF + thrombocytopenia	Carica papaya	Mean increase ± SD	4	+106 ± 69.16 vs 82.35 ± 37.28	142.26 ± 74.63 vs 116.50 ± 39.187
Ganjhu 2022 [23]	DF + thrombocytopenia	Ganjhuvirr	Mean ± SD	5	+87.29 vs 41.04	159.19* ± 18.22 vs 102.10* ± 13.19
Kumar 2024 [24]	DF	Doxycycline	Median (IQR)	4	+174.5 vs + 194.5	190.5 (143–275) vs 211 (100–318)
Pambhar 2022 [27]	DF + thrombocytopenia	Doxycycline + carica papaya or doxycycline	Mean ± SD	7	+145.1 vs + 104.7 vs + 66.0	179.3* ± 26.8 vs 163.8* ± 24.1 vs 134.7* ± 25.3
Sathyapalan 2020 [28]	DF + thrombocytopenia	Carica papaya	Mean percentage increase ± SD	3	482% ± 284* vs 331% ± 370	110.58 vs 94.82
Soroy 2014 [29]	DHF	Propoelix^TM^	Mean ± SD	6	+53.62 vs + 9.51	146.67* ± 64.68 vs 107.84 ± 57.22
Subenthiran 2013 [30]	DF or DHF	Carica papaya	Mean	2	+15.0 vs + 5.0	79.6 vs 69.4*
Tunjungputri 2022 [33]	DF + thrombocytopenia	Oseltamivir	Median (IQR) change from baseline	2	−3 (−16 – +15.5) vs + 17 (−5.5 – +44.75)	41 vs 60
Yang 2023 [35]	DF	CSJD granules	Mean ± SD	5	+17.2 vs + 48.0	144.2 ± 66.6 vs 159.0 ± 87.0

*Significant value for the difference between intervention and control groups (*p* < 0.05).

Abbreviations: DF = dengue fever; DHF = dengue hemorrhagic fever; SD = standard deviation; IQR = interquartile range; CSJD = Chai-Shi-Jie-Du.

Doxycycline alone showed a significant difference compared to the control group at 7 days after baseline (168.3 × 10^9^/L ±24.1 versus 134.7 × l10^9^/L ±25.3 (*p* = 0.03)), yet this difference was greater in the combination of doxycycline with *Carica papaya* (197.3 × 10^9^/L ±26.8 (*p* = 0.04) [30]. This study allocated patients to the different study arms based on their platelet counts. Patients assigned to receive doxycycline in combination with *Carica papaya* had the lowest platelet count on day 0, yet showed the biggest rise in platelet count by day 7. Another study that examines doxycycline alone did not show a significant rise in platelet count [27].

#### Value of IL-6

Four articles documented data regarding the value of IL-6 [24,25,27,31]. The outcome characteristics and results are recorded in Table 5. Three of the studies used doxycycline as intervention [24,25,27], two of which found a significant result [24,25]. Castro *et al*. showed a significant reduction in IL-6 for both the doxycycline and tetracycline groups, in patients with DF as well as patients with DHF [24]. This decrease was greater in patients with DF, with endpoint values of 3.96 in the doxycycline group, 5.48 in the tetracycline group, and 7.41 in the control group (*p* < 0.01). No difference could be found with the use of *Carica papaya* [31].

**Table 5.** Data on the value of IL-6 (in pg/mL).

Study	Patient population	Intervention	Reported as	Time of endpoint (in days)	Change from baseline	Value at endpoint
Castro 2011 [21]	DF	Doxycycline or tetracycline	Mean	7	−3.51 vs −1.9 vs + 0.42	3.96* vs 5.48* vs 7.41
	DHF	Doxycycline or tetracycline	Mean	7	−3.18 vs −0.88 vs + 0.45	2.36* vs 4.23* vs 5.20
Fredeking 2015 [22]	DHF	Doxycycline	Mean	7	−2.99 vs −0.95	2.99* vs 4.49*
Kumar 2024 [24]	DF	Doxycycline	Median (IQR)	4	−19.75 vs −14.53	1.95 (0.57–7.28) vs 0.57 (0.00–8.32)
Sathyapalan 2020 [28]	DF + thrombocytopenia	Carica papaya	Mean ± SD	3	−4.16 vs −2.65	18.80 ± 9.92 vs 22.92 ± 4.07

*Significant value (*p* < 0.05).

Abbreviations: DF = dengue fever; DHF = dengue hemorrhagic fever; IQR = interquartile range; SD = standard deviation.

#### NS1 clearance time

Three studies reported on the NS1 clearance time [29,34,35]. This can be seen in Table 6. Studies with this outcome included patients with DF at baseline. Ivermectin treatment resulted in a shorter NS1 clearance time of 71.5 hours (95% CI 59.9–84.0) compared to the control group, which reported a clearance time of 95.8 hours (95% CI 83.9–120.0) [34]. For balapiravir and chloroquine however, this effect could not be demonstrated [29,35].

**Table 6.** Data on NS1 clearance time (in hours).

Study	Inclusion criteria	Intervention	Reported as	Result (intervention vs control)
Nguyen 2013 [26]	DF	Balapiravir	Median (IQR)	96 [72-144] vs 96 [72-312]
Suputtamongkol 2021 [31]	DF	Ivermectin	Mean (95% CI)	71.5* (59.9–84.0) vs 95.8 (83.9–120.0)
Tricou 2010 [32]	DF	Chloroquine	Median (IQR)	96 (65.5–115) vs 94.5 [48-120]

*Significant value (*p* < 0.05).

Abbreviations: DF = dengue fever; IQR = interquartile range; CI = confidence interval.

#### Virological log reduction

Table 7 presents data on the virological log reduction (in log_10_ copies/mL/day). The studies included in this table provide the mean reduction in viral load along with the standard deviation (SD) for both the intervention and control groups. Both studies included patients diagnosed with DF [25,31].

**Table 7.** Data on viral log reduction (in log_10_ copies/mL/day).

Study	Inclusion criteria	Intervention	Reported as	Result (intervention vs control)
Low 2014 [25]	DF	Celgosivir	Mean ± SD	−1.68 ± 1.07 vs −1.64 ± 0.75
Suputtamongkol 2021 [31]	DF	Ivermectin	Mean ± SD	−2.38 ± 1.16 vs −2.45 ± 1.27

Abbreviations: DF = dengue fever; SD = standard deviation.

Patients treated with celgosivir showed a slightly greater reduction of the viral load compared to controls, whereas patients treated with ivermectin appear to have a smaller decrease in viral load compared to the control group. However, both these findings were not significant [25,31].

### Immunomodulatory interventions

#### Corticosteroids

Three studies focused on corticosteroids [41,47,48]. Two of these, investigated the effect of intravenous dexamethasone in patients with DF and thrombocytopenia [41,47].

Kularatne *et al*. administered a dose of 10 mg, starting with 4 mg followed by 2 mg every 8 hours for the remaining 24 hours [41]. Shashidhara *et al*. used a higher dosing regimen, starting with 8 mg followed by 4 mg three times daily for four days [47]. In both trials, no significant difference in platelet count was found between the intervention and control groups after four days [41,47].

Shashidhara *et al*. also stated a similar hospital stay for both groups, although no specific numbers were provided [47].

Tam *et al*. investigated the effect of oral prednisolone, both in a low dose (0.5 mg/kg/day), and in a high dose (2 mg/kg/day) over a three-day course. The study could not demonstrate a significant difference in NS1 clearance time, which were comparable across the high-dose, low-dose, and control groups [48].

#### Antihistamines

Four studies assessed the effect of antihistamines, focusing on either montelukast [40,44], or on rupatadine [42,43].

Montelukast is a leukotriene receptor antagonist and has been shown to reduce plasma leakage in animal studies [49]. Two studies that tested this agent in patients with DF were recently published and had a relatively large sample size [40,44]. This prospective cohort study [40] and RCT [44] examined length of hospital stay as an outcome. Dev *et al*. [40] showed that the montelukast group had a shorter mean hospital stay of 4.52 ± 1.91 days compared to the non-montelukast group, which had a mean hospital stay of 6.54 ± 2.50 days (*p* < 0.001). However, Nitinai *et al*. [44] were unable to confirm this difference in an RCT, as the median hospital stay was 3 days both in the intervention and control groups (*p* = 0.478).

Rupatadine has blocking activity on the platelet activating factor (PAF) receptor, which presence is associated with vascular leakage during DENV-infection [50,51]. Use of Rupatidine showed a more rapid rise in platelet count in two RCT’s. Malavige *et al*. demonstrated a median difference of 70.5 × 10^9^/L (IQR 41.25–92.75) in the intervention group versus a median of 53 x10^9^/L (IQR 29.5–83.5; *p* = 0.01) on day 7 [42]. The second RCT, published in 2022 demonstrated a a mean platelet count of the rupatadine group of 129.77 × 10^9^/L versus 103.20 x10^9^/L in the control group on day 7 (*p* = 0.01).

### Platelet-enhancing agents

Three studies aimed to increase platelet counts by administering platelet-enhancing agents [39,45,46]. Chakrabotry *et al*. [39] evaluated the effect of eltrombopag, a thrombopoietin receptor agonist [52] at doses of 25 mg and 50 mg daily. Pannu *et al*. [45] investigated anti-RhD immunoglobulin (anti-D), which blocks the Fcγ receptor and has previously demonstrated efficacy in improving platelet counts in patients with immune thrombocytopenic purpura (ITP) [53]. Additionally, rhIL-11, which stimulates megakaryopoiesis and thus the platelet production [54], was also assessed.

All studies contained patients with DF and thrombocytopenia and showed a significant increase in platelet counts between the intervention and control groups. With rhIL-11 and anti-D, this effect was already evident after 12 and 24 hours, respectively, while eltrombopag showed a significant effect starting from day 4 [39,45,46]. This increase in platelet count continued for eltrombopag until the end of the trial at day 7, with platelet counts of 332 × 10^9^/L ±92 in the low-dose group and 371 × 10^9^/L ±111 in the high-dose group, versus 194x10^9^/L ±96 in the control group (*p* < 0.001) [39].

Two studies had relatively short follow-up periods of 48 hours after baseline, but the rise in platelet counts remained statistically significant throughout this period. However, one of these studies by Pannu *et al*. reported that platelet counts at discharge were not significantly different between the anti-D and control groups, as was the length of hospital stay with an average of 6 days [45,46].

## Discussion

The aim of this systematic review was to provide an overview of the effectiveness of therapeutic agents for the treatment of DF. To our knowledge, this is the first systematic review to synthesize evidence from adult and mixed adult – pediatric trials, while excluding pediatric-only studies to preserve clinical homogeneity.

The main part of the results focused on agents with antiviral activity. The (theoretical) evidence supporting this antiviral activity was established either in the included studies, or in studies not eligible for this systematic review. *In vitro* studies showed inhibition of DENV for celgosivir, balapiravir, doxycline, chloroquine, ivermectin, and CSJD granules [29,55–59]. The evidence of DENV-inhibition for lovastatin was investigated in an *in vivo* study [60]. Oseltamivir inhibits neuraminidase, an enzyme involved in viral release from host cells, and is widely used for the prevention and treatment of influenza [61]. The use of herbal extracts, like *Carica papaya* herbal extract is gaining more interest. Therefore, in this systematic review, *Carica papaya* was well represented as an intervention, with studies investigating its effect on hospital stay, platelet count, and levels of IL-6. Ganjhuvirr previously demonstrated antiviral and anti-inflammatory effects [62]. This antiviral effect was confirmed in the study of Ganjhu *et al*., included in this review, through a significant improvement in VLR [26]. The clinical relevance of Ganjhuvirr is further emphasized by the observed shorter duration of hospitalization. *Carica papaya* showed *in vitro* anti-DENV activity by significantly diminishing both the intracellular viral load as also the NS-1 protein expression. Sathyapalan *et al*. investigated this effect on IL-6 values, reporting substantially higher values compared to those observed in other studies included in this review. This may be attributed to the study setting. The study conducted by Sathyapalan *et al*. took place in a tertiary care hospital, where more complex care for the severely ill is given. Therefore, it is reasonable to assume that IL-6 levels in this population were higher compared to those in the other studies [31]. Although promising biochemical results were observed in this tertiary care population, no robust clinically relevant differences were found in the duration of hospital stay. Propoelix^TM^ was found to exhibit only anti-inflammatory effects [63]. For both hospital stay and platelet count, some studies found a significant outcome, whereas other studies failed to find a significant improvement in the primary outcome. These conflicting results have also been observed with the use of doxycycline [21,22,24,27] and montelukast [40,44]. Therefore, there is still inconclusive evidence for the effectiveness of these interventions as a treatment option for DENV infection.

This systematic review also focused on immunomodulatory and platelet enhancing agents. We included three trials that assessed the use of corticosteroids in a non-pediatric population of DF patients. No significant difference was found regarding platelet count and NS1 clearance time. This is in accordance with a previous systematic review, that showed no evidence of corticosteroids on mortality and disease progression in children and adults [64]. Doxycycline however, has shown immunomodulatoy capabilities as well by playing a role in the regulation of matrix metalloproteinases (MMPs). This is due to MMP-9, which has been thought to play a role in capilary leakage in DSS when upregulated by DENV [65,66].

Some of the studies investigating platelet-enhancing agents appear promising, demonstrating a significant rise in platelet count with eltrombopag [39], anti-D [45], and rhIL-11 [46]. However, it is crucial to also focus on clinical outcomes such as bleeding manifestations, plasma leakage, and mortality. In addition to thrombocytopenia, partly due to bone marrow suppression and binding of platelets to leukocytes, alterations in platelet function also occur following a DENV infection. These changes further impact the severity of the disease course [67–69]. Above that, *Carica papaya* is also thought to have thrombopoietic properties and has therefore also been investigated in other diseases with thrombocytopenia, such as chronic immune thrombocytopenic purpura (ITP). Carpaine, an alkaloid being the major component of *Carica papaya*, is thought to be responsible for this platelet augmention. The mechanism of action on thrombopoiesis has to lay in induction of thrombopoietin (TPO) via the TPO-receptor and induction of IL-6 [70,71]. When combined with Doxycycline, this thrombopoietic effect is more pronounced. This synergistic effect may be attributed to the distinct yet complementary effector functions of both agents. *Carica papaya* has thrombopoietic properties while Doxycycline has both antiviral and anti-inflammatory effects, resulting in not only improvement of platelet count but also reduced duration of hospitalization. Yet, previous systematic reviews stated that the current evidence lacked the ability to draw unilateral conclusions on the effectiveness of *Carica papaya* as a treatment for DENV [64,65].

In this systematic review, we excluded studies that solely focus on pediatric populations. In addition to the well-studied pharmacokinetic differences between children and adults, the DENV infection itself also plays a role in the possibility of retrieving heterogeneous outcomes [72]. A 2024 study demonstrated that the clinical manifestations of a DENV infection differ between pediatric and adult populations. In children, symptoms such as skin rash, thrombocytopenia, and hypotension are more prominent, whereas adults more frequently experience pain, chills, and tachypnea [73]. As a result, the included interventions may exert contradictory results when assessed only in a pediatric population, complicating the ability to draw definitive conclusions. Therefore, studies with a pediatric and adult population or an adult population solely were considered eligible for inclusion.

When looking at the limitations of this study, many of the included studies had small sample sizes, reducing their statistical power and increasing the risk of biased results. Firstly, clinical heterogeneity is present due to differences in study populations, with study population differing in DF or DHF (now officialy stated as dengue with or without warning signs or SD) and the characteristics of the specific intervention differing in both dosage and timing, as the duration of treatment. Although the inclusion was restricted to adolescents and adults with confirmed DENV infection to reduce this clinical variability, some degree of population-level discrepancies remains inevitable. This may also partly explain why studies investigating the same agent, despite primarily assessing similar clinical outcomes, often yielded conflicting results. Meanwhile, robust clinical outcomes, such as the reduction of bleeding events or the prevention of severe dengue, are endpoints of crucial importance. Secondly, methodological heterogeneity was present across studies. The trial design differed greatly between studies as both randomized and non-randomized trials were represented in this analysis. Some studies lacked blinding or did not report allocation concealment, possibly increasing the risk of both performance and detection bias. This limitation, along with consistently low scores in other domains of the quality assessment, negatively impacted the overall quality of the studies. Furthermore, the inconsistent availability of raw data from some authors prevented the possibility of conducting a meta-analysis. Another limitation is the variability in treatment initiation timing. During the course of a DENV-infection, viremia levels rapidly decline following symptom onset, in particular after two days of illness [74]. Antivirals should therefore be administered early in the disease process, which is not always feasible due to patient delay in presentation to a medical facility [75]. As a consequence, it is possible that studies including patients later in the disease course reported a reduced antiviral effect of the intervention investigated. Therefore, trials regarding antiviral agents preferebally are designed in an outpatient setting with the aim of preventing hospitalization. Yet, we consider this review the most complete and recent overview of therapeutic options. This is due to most of the included studies being RCT’s with adequate randomization, minimizing potential selection bias.

Looking forward, multiple new perspectives could contribute to effective DENV treatment development. A key example are monoclonal antibodies (MAbs). VIS513, a humanized Mab targeting domain III of the envelope protein, show promising results. In animal models, VIS513 was able to cause robust neutralization of all DENV serotypes and above that, was capable of overcoming ADE of infection by robustly diminishing viremia. Therefore, in 2024 a phase one study in humans was executed to investigate the performance of VIS513. Although this was a small study, these preliminary results showed that the MAb was well tolerated without any serious side effects [76,77]. If proven effective, several questions remain before implementation. Most importantly the risk of ADE, which can also present in cases of seropositivity to other flaviviruses before getting infected with DENV [78]. Next to that, Mab are only effective in the relatively short viremic phase of infection. In addition, it is necessary to prioritize the investigation of clinical effectiveness of mAbs against all 4 DENV serotypes [79]. An alternative method for finding new therapeutics is drug repurposing. With this approach, already approved agents, with or without the use of algorithms, are investigated for their antiviral activity against DENV [80]. The advantage being safety and tolerability assessments have been previously conducted, allowing for the acceleration of research into potential antiviral effects. While promising, they have been published relatively recently, and RCT’s are yet to demonstrate the actual effect of these agents in patients with DF [81–84].

## Conclusion

In conclusion, no effective treatment modality has been objectivated yet, due to conflicting results and an overall moderate to low quality of the included studies. To illuminate more concise results, large RCTs are needed, particularly focusing on doxycycline, montelukast, rupatadine, *Carica papaya*, and platelet enhancing agents with a primary emphasis on clinically relevant endpoints.

## Supplementary Material

Suplementary File 1.docx

## Data Availability

The extra analyses done were exhibited with raw data provided by the original authors of the selected studies when necessary to assess the outcome. These data are available at https://doi.org/10.6084/m9.figshare.28194947.
