# Supplementary material for: Effectiveness of antiviral, immunomodulatory and platelet-enhancing agents for treatment of dengue infection: A systematic review
Source: Virulence. 2025 Nov 10;16(1):2587491. doi: 10.1080/21505594.2025.2587491 (PMC12622339; doi:10.1080/21505594.2025.2587491)
Supplement: Suplementary File 1.docx [file KVIR_A_2587491_SM5680.docx]

# Suplementary File 1: Search strings and corresponding results

| **Database searched** | **Platform** | **Years of coverage** | **Records** | **Records after duplicates removed** |
| --- | --- | --- | --- | --- |
| Medline ALL | Ovid | 1946 - Present | 677 | 667 |
| Embase | Embase.com | 1971 - Present | 1563 | 1043 |
| Web of Science Core Collection* | Web of Knowledge | 1975 - Present | 1620 | 1105 |
| Cochrane Central Register of Controlled Trials | Wiley | 1992 - Present | 353 | 145 |
| **Total** | | | **4213** | **2960** |

**Embase 1563**

(dengue/exp OR (dengue* OR ((DENV) NEAR/3 (infect*))):ab,ti,kw) **AND** (therapy/exp OR therapy:lnk OR ‘antivirus agent’/exp OR ‘antiviral therapy’/exp OR 'antiinfective agent'/exp OR 'antibiotic agent'/exp OR 'antiparasitic agent'/exp OR (therap* OR treat* OR antivir* OR anti-vir* OR antiinfectiv* OR anti-infectiv* OR antibiotic OR anti-biotic OR antibacterial OR anti-bacterial OR antimicrobial OR anti-microbial OR antiparasitic OR anti-parasitic OR oseltamivir* OR lovastatin* OR antiinfectives OR anti-infectives OR ivermectin* OR doxycyclin* OR chloroquin*):ab,ti,kw) **AND** ('Controlled clinical trial'/exp OR 'Crossover procedure'/de OR 'Double-blind procedure'/de OR 'Single-blind procedure'/de OR (random* OR factorial* OR crossover* OR (cross NEXT/1 over*) OR placebo* OR ((doubl* OR singl*) NEXT/1 blind*) OR assign* OR allocat* OR volunteer* OR trial OR groups):ab,ti,kw) NOT ((animal/exp OR animal*:de OR nonhuman/de OR 'in vitro study'/de OR (in-vitro OR invitro):ab,ti) NOT ('human'/exp)) NOT (vaccine/mj/exp OR (vaccin* OR in-vitro* OR invitro*):ti)

**Medline 677**

(exp Dengue/ OR (dengue* OR ((DENV) ADJ3 (infect*))).ab,ti,kf.) **AND** (therapy.fs. OR exp Therapeutics/ OR exp Antiviral Agents/ OR exp Anti-Infective Agents/ OR exp Anti-Bacterial Agents/ OR exp Antiparasitic Agents/ OR (therap* OR treat* OR antivir* OR anti-vir* OR antiinfectiv* OR anti-infectiv* OR antibiotic OR anti-biotic OR antibacterial OR anti-bacterial OR antimicrobial OR anti-microbial OR antiparasitic OR anti-parasitic OR oseltamivir* OR lovastatin* OR antiinfectives OR anti-infectives OR ivermectin* OR doxycyclin* OR chloroquin*).ab,ti,kf.) **AND** (exp Controlled Clinical Trial/ OR exp Cross-Over Studies/ OR Double-Blind Method/ OR Single-Blind Method/ OR (random* OR factorial* OR crossover* OR (cross ADJ over*) OR placebo* OR ((doubl* OR singl*) ADJ blind*) OR assign* OR allocat* OR volunteer* OR trial OR groups).ab,ti,kf.) NOT (exp Animals/ NOT Humans/) NOT (exp *Vaccines/ OR (vaccin* OR in-vitro* OR invitro*).ti.)

**Cochrane 353**

((dengue* OR ((DENV) NEAR/3 (infect*))):ab,ti,kw) **AND** ((therap* OR treat* OR antivir* OR anti-vir* OR antiinfectiv* OR anti-infectiv* OR antibiotic OR anti-biotic OR antibacterial OR anti-bacterial OR antimicrobial OR anti-microbial OR antiparasitic OR anti-parasitic OR oseltamivir* OR lovastatin* OR antiinfectives OR anti-infectives OR ivermectin* OR doxycyclin* OR chloroquin*):ab,ti,kw) NOT ((vaccin* OR in-vitro* OR invitro*):ti)

**Web of Science 1620**

TS=(((dengue* OR ((DENV) NEAR/2 (infect*)))) **AND** ((therap* OR treat* OR antivir* OR anti-vir* OR antiinfectiv* OR anti-infectiv* OR antibiotic OR anti-biotic OR antibacterial OR anti-bacterial OR antimicrobial OR anti-microbial OR antiparasitic OR anti-parasitic OR oseltamivir* OR lovastatin* OR antiinfectives OR anti-infectives OR ivermectin* OR doxycyclin* OR chloroquin*)) **AND** (random* OR factorial* OR crossover* OR (cross NEAR/1 over*) OR placebo* OR ((doubl* OR singl*) NEAR/1 blind*) OR assign* OR allocat* OR volunteer* OR trial OR groups)) NOT TI=(vaccin* OR in-vitro* OR invitro*)
